# Regional Brain Structural Abnormality in Meal-Related Functional Dyspepsia Patients: A Voxel-Based Morphometry Study

**DOI:** 10.1371/journal.pone.0068383

**Published:** 2013-07-02

**Authors:** Fang Zeng, Wei Qin, Yue Yang, Danhua Zhang, Jixin Liu, Guangyu Zhou, Jinbo Sun, Shengfeng Lu, Yong Tang, Yuan Chen, Lei Lan, Shuguang Yu, Ying Li, Xin Gao, Qiyong Gong, Jie Tian, Fanrong Liang

**Affiliations:** 1 The 3rd Teaching Hospital, Chengdu University of Traditional Chinese Medicine, Chengdu, Sichuan, China; 2 Life Sciences Research Center, School of Life Sciences and Technology, Xidian University, Xi’an, Shaanxi, China; 3 Psychosomatic Medicine Department, Sichuan Academy of Medical Sciences & Sichuan Provincial People’s Hospital, Chengdu, Sichuan, China; 4 Key Laboratory of Acupuncture and Medicine Research of Minister of Education, Nanjing University of Traditional Chinese Medicine, Nanjing, Jiangshu, China; 5 Biology Department, University of Pennsylvania, Philadelphia, Pennsylvania, United States of America; 6 Department of Radiology, The Center for Medical Imaging, Huaxi MR Research Center, West China Hospital of Sichuan University, Chengdu, Sichuan, China; 7 Institute of Automation, Chinese Academy of Sciences, Beijing, China; Charité University Medicine Berlin, Germany

## Abstract

**Background and Aims:**

Brain dysfunction in functional dyspepsia (FD) has been identified by multiple neuroimaging studies. This study aims to investigate the regional gray matter density (GMD) changes in meal-related FD patients and their correlations with clinical variables, and to explore the possible influence of the emotional state on FD patients’s brain structures.

**Methods:**

Fifty meal-related FD patients and forty healthy subjects (HS) were included and underwent a structural magnetic resonance imaging scan. Voxel-based morphometry analysis was employed to identify the cerebral structure alterations in meal-related FD patients. Regional GMD changes' correlations with the symptoms and their durations, respectively, have been analyzed.

**Results:**

Compared to the HS, the meal-related FD patients showed a decreased GMD in the bilateral precentral gyrus, medial prefrontal cortex (MPFC), anterior cingulate cortex (ACC) and midcingulate cortex (MCC), left orbitofrontal cortex (OFC) and right insula (*p*<0.05, FWE Corrected, Cluster size>50). After controlling for anxiety and depression, the meal-related FD patients showed a decreased GMD in the bilateral middle frontal gyrus, left MCC, right precentral gyrus and insula (*p*<0.05, FWE Corrected, Cluster size>50). Before controlling psychological factors, the GMD decreases in the ACC were negatively associated with the symptom scores of the Nepean Dyspepsia Index (NDI) (r = −0.354, *p* = 0.048, Bonferroni correction) and the duration of FD (r = −0.398, *p* = 0.02, Bonferroni correction) respectively.

**Conclusions:**

The regional GMD of meal-related FD patients, especially in the regions of the homeostatic afferent processing network significantly differed from that of the HS, and the psychological factors might be one of the essential factors significantly affecting the regional brain structure of meal-related FD patients.

## Introduction

Functional dyspepsia (FD) is defined by the presence of one or more of four chronic symptoms (epigastric pain, epigastric burning, early satiation and postprandial fullness) in the absence of any organic, systemic or metabolic disease that is likely to explain the symptoms [1]. According to the Rome III criteria, FD includes two distinct syndromes: (1) postprandial distress syndrome (PDS, or meal-related FD), consisting of bothersome postprandial fullness and/or early satiation, and (2) epigastric pain syndrome (EPS, or meal-unrelated FD), consisting of pain or burning sensation localized in the epigastric area [2]. PDS is much more common than EPS among FD patients.

As a highly prevalent functional gastrointestinal disease (FGID), the pathophysiology of FD has been widely investigated in the past two decades. Although several theories, including gastrointestinal motility abnormalities, impaired gastric accommodation, visceral hypersensitivity, *Helicobacter pylori* infection, altered response to duodenal lipids or acid and psychological factors have been proposed to elucidate the symptoms, the underlying cause of FD remains uncertain [3]. With the application of neuroimaging techniques in the research of gastrointestinal disorders, there is an increasing body of evidence pointing to the importance of the Central Nervous System (CNS) ’s influence on the pathology of FGID including FD [4]. In 2007, Vandenberghe and his colleagues conducted a positron emission tomography (PET) study on the FD patients with symptoms of hypersensitivity. The results demonstrated that, compared to the healthy subjects (HS), FD patients showed activations in the bilateral gyrus precentralis, gyrus frontalis inferior, gyrus frontalis medialis, gyrus temporalis superior, cerebellar hemisphere and the left gyrus temporalis inferior at significantly lower distention pressures [5]. In 2010, Van Oudenhove L, *et al*. reported that the brain processing of gastric distention stimuli as well as sham distension in FD patients differed from those in the HS, independent of the hypersensitivity. They also found that anxiety correlated negatively with the pregenual anterior cingulate cortex (pACC) and middle cingulate cortex (MCC), while positively with dorsal pons activity [6], and that abuse history was associated with differences in the insula, prefrontal, and hippocampus/amygdala activity [7]. In a previous study, using Fluorine-18 Fluorodeoxyglucose (18F-FDG) PET-CT, we found that FD patients showed extensively increasing cerebral glycometabolism especially in the homeostatic afferent processing network, and increased cerebral glycometabolism in the ACC, MCC, insula, thalamus and cerebellum which were positively related to symptom severity of FD [8]. All of these studies demonstrated the differences in brain activities between the FD patients and the HS, and indicated that the pathophysiology of FD involved cerebral dysfunction.

Recently, structural magnetic resonance imaging to investigate brain anatomical abnormalities has been used actively to uncover the pathogenesis of diseases. Some functional gastroenterology studies [9]–[12] have investigated the brain structural changes in irritable bowel syndrome (IBS). For example, Blankstein U, *et al*. [10] found that the IBS patients showed increased hypothalamic gray matter and cortical thinning in the anterior midcingulate cortex compared with controls. Seminowicz DA, *et al*. [11] proved that IBS was associated with decreased gray matter density (GMD) in the medial prefrontal cortex (MPFC), ventrolateral prefrontal cortex, posterior parietal cortex, ventral striatum and thalamus, and increased GMD in the pACC and the orbitofrontal cortex (OFC). These studies provided a new approach to exploring the pathogenesis of FGID. However, no study has been performed on the FD patients.

We hypothesize that the persistent and recurrent dyspepsia experience and the significant cerebral functional abnormality might influence the brain structure of FD patients. By using structural fMRI, this study aimed to 1) compare the cerebral GMD difference between the meal-related FD patients and the HS with a relatively large and tightly screened sample; 2) explore the possible influence of the emotional state on regional GMD and 3) investigate the correlations of regional GMD changes with clinical variables.

## Participants and Methods

### Participant Selection

50 right-handed meal-related FD patients (18 males) and 40 age-matched, right-handed HS (14 males) were enrolled in this study. All of these participants signed a written informed consent form.

The FD patients were recruited at the outpatient department in the Teaching Hospital of Chengdu University of Traditional Chinese Medicine from April. 2011 to Oct. 2011. Each patient was evaluated by 2 gastroenterologists and a psychologist, and underwent careful laboratory examinations including upper gastrointestinal endoscopy, upper abdominal ultrasound, electrocardiogram, hepatic function, renal function and routine analysis of blood, urine and stool. The inclusion criteria required that all of the patients be 20 to 30 years of age, match the Rome III criteria on FD and the Rome III criteria on PDS. Patients were screened out if they: 1) were or might be pregnant, or were lactating, or 2) were suffering from or had a history of serious neurological, cardiovascular, respiratory or renal illnesses, or 3) had a history of gastrointestinal surgery, dysmenorrhea, diabetes or head trauma with loss of consciousness, or 4) were suffering from mental disorders including major depressive disorder, anxiety disorder, bipolar disorder, schizophrenia, claustrophobic syndrome, or 5) had been using aspirin, non-steroidal anti-inflammatory drugs, steroids, phenothiazines, selective serotonin reuptake inhibitors or medication affecting gastrointestinal motility for over 2 weeks before enrollment, or 6) were currently participating in other clinical trials.

The HS were recruited by advertisement. Each HS was free from any gastrointestinal symptoms or signs, and underwent a basic evaluation, including a review of medical history, a physical examination, gastrointestinal endoscopy, upper abdominal ultrasound and electrocardiogram.

This study was performed according to the principles of the Declaration of Helsinki. The study protocol was approved by the Ethics Committee of Chengdu University of Traditional Chinese Medicine (NO.2011KL003).

### Symptom Assessment

The symptom severity and the quality of life (QOL) were assessed by using the Nepean Dyspepsia Index (NDI). NDI is dyspepsia-specific and designed to measure both symptoms and health-related QOL [13]. The symptom score of the NDI is based on 15 dyspepsia-related physical signs rated for frequency (0–4), intensity (0–5) and bothersomeness (0–4). The number 0 represents no symptoms, and higher numbers paralleled worsening of the symptoms. The QOL index of the NDI includes 4 domains, namely interference (13 items), know/control (7 items), eat/drink (3 items) and sleep/disturb (2 items). Higher scores indicate better QOL. The translated version of NDI was found, by our prior research, to be reliable and valid for measuring symptom severity and QOL in Chinese patients with FD [14].

### Psychosocial Evaluation

The Zung Self-Rating Anxiety Scale (SAS) [15] and the Zung Self-Rating Depression Scale (SDS) [16] were employed in this study to quantify the anxiety/depression related symptoms of the participants. Both scales consist of 20 items. Each item is scored from 1 to 4. According to the Chinese norm [17]–[18], an index score of SAS (calculated by multiplying the raw score by 1.25) less than 50 or an index score of SDS (calculated by multiplying the raw score by 1.25) less than 53 falls into the normal range.

### MRI Scan

Brain images were acquired on a 3T Siemens MRI scanner (Allegra; Siemens Medical System) at the Huaxi Magnetic Resonance Research Center, West China Hospital of Sichuan University, Chengdu, China. During the scan, each participant, with eyes blindfolded and ears plugged, underwent a high resolution 3-dimensional T1-weighted, sagittal, magnetization-prepared rapid gradient echo (MPRAGE) sequence (TR = 1900 ms; TE = 2.26 ms; flip angle = 9°; in-plane matrix resolution = 256×256; slices = 176; field of view = 256 mm; voxel size = 1×1×1 mm). For female participants, scanning took place during the week following menstrual period to avoid the possible changes in brain size and activity in menstrual cycles [19]–[20].

### Data Analysis

#### Clinical variables

All the physiological and psychological measures were analyzed using the SPSS 17.0 (SPSS Inc, Chicago, IL) by 2 blinded evaluators. All the numerical variables in this article are presented as mean±standard deviation (SD). Independent-Samples t Test was used on numerical variables. Chi-square test was used on categorical variables and two-sided test was applied on all available data. P value <0.05 was considered statistically significant.

#### VBM analysis

Structural data was processed with an FSL-VBM protocol [21] with FSL 4.1 software [22] (http://www.fmrib.ox.ac.uk/fsl). Firstly, the T1 images were processed with the following two procedures: brain extraction by BET [23] and tissue-type segmentation using FAST 4.1 [24]. The resulting grey-matter partial volume images were then aligned to MNI152 standard space using the FMRIB’s linear image registration tool (FLIRT) [25], followed by FMRIB’s nonlinear image registration tool (FNIRT) [26]–[27] which uses a b-spline representation of the registration warp field [28]. The resulting images were averaged to create a study-specific template, to which the native gray matter images were then nonlinearly re-registered. In order to correct for local expansion or contraction, the registered partial volume images were modulated by dividing by the Jacobian of the warp field. An isotropic Gaussian kernel with a sigma of 3 mm was then used to smooth the modulated segmentated images. Finally, a voxelwise group-difference was assessed by using permutation-based non-parametric testing (Randomise v2.1, 10, 000 permutations) with the covariates: gender+age, gender+age+anxiety+depression. For multiple comparison corrections, the threshold-free cluster enhancement (TFCE) with the family-wise error (FWE) correction was employed [29].

#### The correlation analysis between clinical variables and GMD

In order to assess the relationship between clinical variables and GMD, the mean value of the first ten continuous voxels around the local maximum of each cluster showing a significant group-difference was first extracted. Then the mean values’ correlations with symptom scores of the NDI and disease durations were calculated respectively by using a partial correlation analysis. Bonferroni correction was applied for multiple comparisons. The regions of interest (ROIs) were chosen mainly based on our previous study and the current VBM results. In our previous study [8], we found that the abnormal activities in the ACC, MCC, insula, thalamus and cerebellum were significantly associated with symptom severity of FD patients. In this study, the VBM results without regressing SAS/SDS showed that the GMD in the ACC and in the insula was decreased. So we chose the ACC and the insula as the ROIs for the correlation analysis.

## Results

The data on one meal-related FD patient and one HS were excluded from our analysis due to head motion artifacts.

### Patient Characteristics

Among the meal-related FD patients, the mean symptom score of the NDI was 46.49±15.13; the mean QOL score of the NDI was 77.62±10.15; the mean duration was 36.12±26.89 months; the mean SAS score was 42.07±8.01 and the mean SDS score was 43.27±10.04. According to the SAS and SDS assessment, 12.24% of the meal-related FD patients showed mild anxiety, 20.41% mild depression, and 10.20% both mild depression and anxiety.

There were no significant differences in the demographics including age, sex, weight and height between the meal-related FD patients and the HS (*p*>0.05). There were significant differences in the SAS scores and the SDS scores between the meal-related FD patients and the HS (*p*<0.01) (Table 1).

**Table 1:** The differences in demographic data and emotional state between the FD patients and the healthy subjects.

Items	FD Patients N = 49	Healthy Subjects N = 39	*p* value
Age (y), mean ± SD	22.55±1.78	22.18±0.85	0.202
Sex distribution (% males)	36.73%	35.90%	0.935
Weight (Kg), mean ± SD	51.42±6.74	51.55±7.68	0.931
Height (cm), mean ± SD	162.18±7.70	162.10±7.61	0.961
SAS scores (25–100), mean ± SD	42.07±8.01	33.85±6.40	0.000
SDS scores (25–100), mean ± SD	43.27±10.04	34.81±7.43	0.000

SD, Standard deviation; SAS, Self-Rating Anxiety Scale; SDS, Self-Rating Depression Scale.

### VBM Results

#### VBM results without regressing SAS/SDS

**Figure 1:**
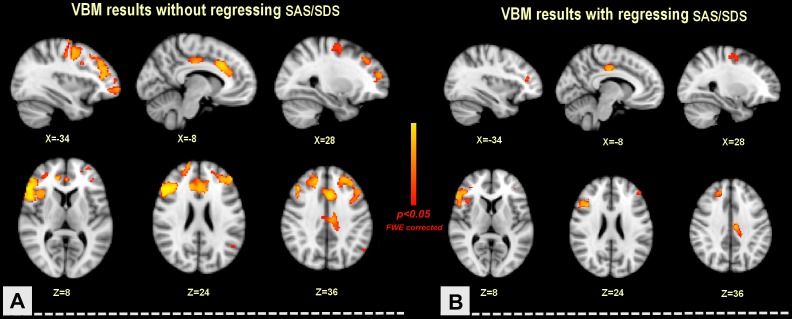
The differences in regional gray matter density between the FD patients and the healthy subjects. Compared to the HS, the FD patients showed a decreased gray matter density (GMD) in the bilateral precentral gyrus, MPFC, ACC and MCC, left OFC and right insula. However, after controlling for both anxiety and depression, GMD decreases in the bilateral middle frontal gyrus, left MCC, right precentral gyrus and insula in the FD group were observed. A: anterior; L: left; p: p value; SAS: Self-Rating Anxiety Scale; SDS: Self-Rating Depression Scale.

Compared to the HS group, the FD group showed GMD decreases in the left OFC (BA10). right insula (BA13), bilateral precentral gyrus (BA6), superior frontal gyrus (BA6, BA 9), middle frontal gyrus (BA9), inferior frontal gyrus (BA46, BA9), ACC (BA32) and MCC (BA32) (*p*<0.05, FWE Corrected, Cluster size>50) (Table 2) (Figure 1).

**Table 2:** The differences in regional gray matter density between the FD patients and the healthy subjects.

Region	Side	Not regress SAS/SDS					Regress SAS/SDS				
		MNI			Cluster Size	BA	MNI			Cluster Size	BA
		X	Y	Z			X	Y	Z		
Precentral Gyrus	L	−28	−8	60	802	BA6	/	/	/	/	/
	R	40	2	42	105	BA6	28	−4	54	56	BA6
Superior Frontal Gyrus	L	−26	−8	60	443	BA6	/	/	/	/	/
	R	18	62	58	588	BA9	22	38	40	133	BA8
Middle Frontal Gyrus	L	−46	22	36	1542	BA9	−48	44	20	90	BA46
	R	48	26	32	582	BA9	24	36	38	128	BA8
OFC	L	−38	60	−6	98	BA10	/	/	/	/	/
Inferior Frontal Gyrus	L	−46	38	12	115	BA46	/	/	/	/	/
	R	48	24	30	1132	BA9	42	20	28	700	BA9
Insula	R	36	20	8	109	BA13	38	22	4	57	BA13
ACC	L	−2	24	30	365	BA32	/	/	/	/	/
	R	14	40	16	493	BA32	/	/	/	/	/
MCC	L	−4	18	34	452	BA32	−10	−14	40	218	BA24
	R	0	24	30	290	BA32	/	/	/	/	/

*p*<0.05, Corrected, Cluster size>50.

Abbreviations: R, right; L, left; BA, Brodmann area; SAS, Self-Rating Anxiety Scale; SDS, Self-Rating Depression Scale; OFC, Orbitofrontal Cortex; ACC, Anterior Cingulate Cortex; MCC, Middle Cingulate Cortex.

#### VBM results regressing SAS/SDS

After controlling for both SAS and SDS, the FD group showed GMD decreases in the bilateral middle frontal gyrus (BA46, BA8), left MCC (BA24) and right precentral gyrus (BA6), superior frontal gyrus (BA8), inferior frontal gyrus (BA9) and insula (BA13), compared to the HS group (*p*<0.05, FWE Corrected, Cluster size>50) (Table 2) (Figure 1).

#### Correlations between GMD changes and clinical variables

The GMD decrease in the insula did not show a significantly negative correlation with the symptom scores of the NDI (*p*>0.05, Bonferroni correction) and the duration of FD (*p*>0.05, Bonferroni correction), and the GMD decrease in the ACC was negatively correlated with the symptom scores of the NDI (r = −0.354, *p* = 0.048, Bonferroni correction) and the duration of FD (r = −0.398, *p* = 0.02, Bonferroni correction) respectively.

After correcting for both SAS and SDS, the structural alterations of the insula were not negatively associated with the symptom scores of the NDI (*p*>0.05, Bonferroni correction) and the duration of FD (*p*>0.05, Bonferroni correction), and the structural alterations of the ACC weren’t associated with symptoms/duration (*p*>0.05, Bonferroni correction).

## Discussion

This study revealed, for the first time, the differences in regional GMD between the meal-related FD patients and the HS, and partly explored the possible reasons for regional GMD changes by correlation analysis, and investigated the influence of psychological factors on cerebral microstructure.

### The Brain Structural Changes in Meal-related FD Patients

With a between-group comparison, we found that the meal-related FD patients showed a decreased GMD in the precentral gyrus, MPFC, ACC, MCC, OFC and insula (Table 2, Figure 1). Our diffusion tensor imaging (DTI) study [30] found that, compared to the HS, the FD patients showed increased fractional anisotropy (FA) along with reduced mean diffusivity (MD) and radial diffusivity (RD) in multiple white matter tracts. These results indicated that the persistent and recurrent discomfort could result in cerebral microstructural changes in the meal-related FD patients, and that although the meal-related FD is currently classified as a somatoform disorder, it is associated with brain structural changes.

Furthermore, in this study, the majority of these regions with GMD changed such as the ACC, insula and PFC, belong to the homeostatic afferent processing network. Our previous PET-CT study [8] indicated that, compared to the HS, the FD patients showed a higher glycometabolism in the regions of the homeostatic afferent processing network especially the ACC, insula and OFC. Our DTI study [30] also demonstrated that FD patients showed altered white matter tracts which were connected with regions of the homeostatic afferent processing network, including right external capsule, right sagittal stratum, right superior longitudinal fasciculus, corpus callosum, corona radiata, right retrolenticular part of the internal capsule, and right posterior thalamic radiata. Thus, we predicted that the structural and functional changes in the homeostatic afferent processing network might be an important character of FD patients.

### The Influence of Psychological Factors on Cerebral GMD

Many studies suggested that psychological factors might be one of the possible causes of FD [31]. It was reported that FD patients had significantly higher levels of psychiatric illness than the HS [32] and the patients with organic dyspepsia [33]. Some studies demonstrated that anxiety seemed to be related to abnormal antral retention of food [34], and that depression was related to the abdominal fullness severity [35]. Furthermore, limited data showed that the meal-related FD patients were more likely to suffer with psychopathology [36]–[37]. Recently, using functional neuroimaging techniques, people found that psychological factors were significantly associated with cerebral dysfunction of FD patients. For example, Van Oudenhove L, *et al*. reported that anxiety was negatively correlated with pACC and MCC, and positively correlated with dorsal pons activity in FD patients [6], and that abuse history was associated with differences in insular, prefrontal, and hippocampus/amygdala activity [7]. In this study, the VBM results indicated that many regions in emotional arousal circuitry of meal-related FD patients showed a significant reduction in GMD. However, after regressing SAS/SDS, the regional GMD changes in some emotion-related regions including the ACC, MCC, MPFC and majority of MCC were not found (Table 2) (Figure 1). Although only part of the FD patients in this study showed mild anxiety and/or mild depression, the SAS scores and the SDS scores of these FD patients were significantly higher than those of the HS (*p* = 0.000). The present results demonstrated that these cerebral microstructural changes in the meal-related FD patients are in part related to the comorbidities of depression and anxiety.

### The GMD Decrease in the Insula and ACC

The insula, considered as one of the key regions of “gut–brain communication”, plays a crucial role in processing and modulating visceral sensory, pain, emotion, and maintaining homeostasis [38]–[39]. Activations in the insula can be found in nearly all reported FGIDs studies, regardless of the study paradigm and analysis methods [40]. Some study [41] demonstrated that the insula processed the interceptive signals of fullness produced by gastric distention. Our previous PET-CT study [8] indicated that, compared to the HS, the meal-related FD patients showed a higher glycometabolism in the insula, and that the abnormal hyperactivity of the insula was significantly related to the symptom severity of FD patients. Furthermore, a MRI study on IBS patients showed a cortical thinning in the insula [9]. In this study, we found that regional GMD in the insula significantly decreased in the meal-related FD patients, and that after correcting SAS/SDS, the decreased GMD of the insula still existed. The results suggested that psychological factors did not have a significant impact on the microstructure of the insula. However, the decreased GMD in the insula didn’t show a significant correlation with durations and symptoms of FD patients no matter whether anxiety and depression were controlled for or not. It indicated that the GMD changes in the insula might be induced by multiple factors and need further investigation.

The ACC is an essential node in the “homeostatic afferent network” and regarded as the “limbic behavioral motor cortex” for its close interconnection with the insula, prefrontal, limbic and other subcortical structures [39]. As an important integration cortex, the functional and structural abnormalities of the ACC in FGIDs have been widely investigated. Similar to the insula, the activation of the ACC was commonly seen in neuroimaging studies on FGIDs [40]. Our previous PET-CT study [8] indicated that cerebral glycometabolism of the ACC in FD patients was significantly higher than that in the HS, and that the abnormal hyperactivity of the ACC was associated with symptoms and QOL of patients. Furthermore, some neuroimaging studies on IBS patients found a cortical thinning and a GMD increase in the ACC [10], [11]. In this study, we found that regional GMD in the bilateral ACC of the meal-related FD patient significantly decreased compared to the HS, and that the GMD decrease in the ACC was negatively correlated with the scores of symptoms and disease duration respectively. However, after correcting for depression and anxiety, the peak of GMC decrease within the ACC did not survive. Our result are consistent with Seminowicz DA’ s findings [11]. His study on IBS patients also showed that inclusion of anxiety and depression together as covariates removed the group differences in the left pregenual ACC. A recent meta-analysis indicated that gray matter reduction in the ACC was the most consistent finding in VBM studies of major depressive disorder (MDD) [42]. The present results indicated that although the ACC is involved in multiple functions, such as the gastrointestinal signal process and emotional and cognitive controls, the anatomic alterations in the ACC in the meal-related FD patients were more likely to be associated with emotional changes and could be attributed neither to the durations nor to the symptoms.

### Conclusions

In summary, this study demonstrated the cerebral morphometric alterations in the meal-related FD patient and the influence of psychological factors on the regional brain structure. Although the majority of the structure-changed regions were related to emotional and cognitive processes, psychosocial dysfunction could not fully explain the microstructural alterations. The cerebral microstructural changes in the meal-related FD patients might be induced by multiple factors, including abnormal sensory input and psychosocial dysfunction, etc. Although the current study was not a longitudinal design and didn’t investigate the causality between the symptoms, psychological factors, duration and altered cerebral structures, the demonstration of gray matter morphometric alterations in the meal-related FD patients could provide a new approach to future studies and give direction to new therapy development.
